# Effects of thermal acclimation on the proteome of the planarian *Crenobia alpina* from an alpine freshwater spring

**DOI:** 10.1242/jeb.244218

**Published:** 2022-08-11

**Authors:** Joshua Niklas Ebner, Mirjam Kathrin Wyss, Danilo Ritz, Stefanie von Fumetti

**Affiliations:** 1 Spring Ecology Research Group, Department of Environmental Sciences, University of Basel, 4056 Basel, Switzerland; 2 Proteomics Core Facility, Biozentrum, University of Basel, 4056 Basel, Switzerland

**Keywords:** Phenotypic plasticity, Proteomics, Molecular adaptation, *Crenobia alpina*, Aquatic ectotherm, Thermal tolerance

## Abstract

Species' acclimation capacity and their ability to maintain molecular homeostasis outside ideal temperature ranges will partly predict their success following climate change-induced thermal regime shifts. Theory predicts that ectothermic organisms from thermally stable environments have muted plasticity, and that these species may be particularly vulnerable to temperature increases. Whether such species retained or lost acclimation capacity remains largely unknown. We studied proteome changes in the planarian *Crenobia alpina*, a prominent member of cold-stable alpine habitats that is considered to be a cold-adapted stenotherm. We found that the species' critical thermal maximum (CT_max_) is above its experienced habitat temperatures and that different populations exhibit differential CT_max_ acclimation capacity, whereby an alpine population showed reduced plasticity. In a separate experiment, we acclimated *C. alpina* individuals from the alpine population to 8, 11, 14 or 17°C over the course of 168 h and compared their comprehensively annotated proteomes. Network analyses of 3399 proteins and protein set enrichment showed that while the species' proteome is overall stable across these temperatures, protein sets functioning in oxidative stress response, mitochondria, protein synthesis and turnover are lower in abundance following warm acclimation. Proteins associated with an unfolded protein response, ciliogenesis, tissue damage repair, development and the innate immune system were higher in abundance following warm acclimation. Our findings suggest that this species has not suffered DNA decay (e.g. loss of heat-shock proteins) during evolution in a cold-stable environment and has retained plasticity in response to elevated temperatures, challenging the notion that stable environments necessarily result in muted plasticity.

## Abstract (precis)

**Summary:** The proteome of an alpine *Crenobia alpina* population shows plasticity in response to acclimation to warmer temperatures.

## INTRODUCTION

Species adapted to cold-stable climates will experience novel selection pressures in response to emerging thermal regimes (Atkins and Travis, 2010; Kenkel et al., 2013). Temperature directly affects the properties of macromolecules, and imposes strong constraints on organisms by affecting the kinetics of chemical reactions. For example, given the rapid decrease in the freedom of molecular motion that accompanies cold temperatures, proteins executing essential cellular processes such as protein synthesis are constrained in their ability to function appropriately (Somero, 1995; 1996). Despite these constraints, life has colonized a wide variety of thermal environments, ranging from volcanic hot vents to thermally stable freshwater habitats. Generating a mechanistic basis for how temperature affects physiological processes and whether organisms from such thermally stable habitats have lost the capacity to compensate for the effects of increasing temperature is becoming more critical (Franklin and Hoppeler, 2021). In concert with adaptive evolution, phenotypic and physiological plasticity is often posited as a central feature of the acute and evolutionary response to climate change (Chevin et al., 2013; Seebacher et al., 2015). The capacity for plasticity, however, depends on the existence of mechanisms to sense and respond to the thermal environment. Both of these mechanisms may either fail to evolve or secondarily be lost in species from thermally stable habitats (Janzen, 1967; Kelly, 2019). As a result, these species are more likely to have narrow ranges of thermal tolerance (i.e. decreased plasticity), but also to exhibit reduced genetic variation in thermal tolerance, making them less likely to evolve wider tolerance over time relative to their eurythermic counterparts (Kelly, 2019). Knowing how species inhabiting thermally stable habitats will respond to rising temperatures is therefore a prerequisite if we are to understand future population dynamics and shifts in community composition (Reusch, 2014; Lamprecht et al., 2018; Rogora et al., 2018). However, there is limited evidence for a relationship between local extinction and limited tolerance to higher temperature (Cahill et al., 2013). The absence of this relationship may be due to the fact that certain organisms from high-elevation habitats are able to maintain homeostasis and stable molecular systems even at higher temperatures than they experience natively (Bernabò et al., 2011, 2020; Hotaling et al., 2020). A variety of molecular responses stabilize and maintain core functional processes such as metabolism, translation, transcription, DNA repair, protein folding, clearance of damaged proteins, mitigation of oxidative stress and regulation of intracellular transport (Thieringer et al., 1998; Morimoto, 2011; De Maayer et al., 2014; Tomanek, 2014). The functional execution and maintenance of these processes results not only from the presence or absence of potential molecular players encoded in the genome, or from stochastic changes in gene expression (McAdams and Arkin, 1997; Elowitz et al., 2002; Raj and van Oudenaarden, 2008), but also from multiple layers beyond the genome (Bludau and Aebersold, 2020), such as the proteome, interactome and whole-organism thermal responses, warranting their study to gain insight into molecular changes close to the phenotype (Feder and Walser, 2005; Karr, 2008).

Remnant populations of certain cold-stenothermal species experienced postglacial retractions and left behind populations in enclaves under relatively benign conditions (Hampe and Jump, 2011; Woolbright et al., 2014). Many of these alpine species are uniquely adapted to cold-stable conditions (Füreder, 1999; Lencioni et al., 2009), including the alpine freshwater flatworm *Crenobia alpina* (Dana 1766), which supposedly has limited thermal tolerance (Voigt, 1895; Thienemann, 1950). The species occurs primarily where annual average water temperature is <15°C and tolerates temperatures as low as 0.7°C (Voigt, 1895; Reynoldson, 1981; Durance and Ormerod, 2007). Despite this preference for the cold habitat patches of rivers and springs, previous investigations also showed a tolerance to a maximum temperature of 25°C for 48 h without adverse effects (Schlieper and Halsband, 1952) and survival in mountain environments with temperature peaks of 25°C (Pattee, 1965), challenging the classification of this species as a cold-adapted stenotherm.

To arrive at a mechanistic understanding of this potential acclimation capacity of *C. alpina*, and to test whether the species exhibits decreased thermal plasticity, we provide a detailed analysis of how its proteome changes following acclimation to warmer temperatures. Using an alpine *C. alpina* population directly from their natal freshwater spring as a model system, we compared the abundance of 3399 proteins after 168 h of acclimation at 8, 11, 14 or 17°C and tested whether sets of temperature-responsive proteins have distinct functional profiles. In a separate experiment, we compared the critical thermal maxima (CT­_max_­) of individuals from three *C. alpina* populations originating from springs with distinct altitudes (526, 1080 and 1548 m.a.s.l.) and temperatures during sampling (11, 9 and 8°C), to test whether there are interpopulational differences in CT_max_ and CT_max_ acclimation capacity.

## MATERIALS AND METHODS

### Determining CT_max_

In all sampling instances, individuals were collected with a 2 or 5 ml pipette and transferred into a cooling box containing water from the respective spring. Animals were transported to the laboratory within 3 h in a mobile fridge. We compared CT_max_ between one alpine and two non-alpine *C. alpina* populations by collecting similarly sized individuals from three springs (Fig. S1). Although temperature loggers were placed to determine the thermal breadths of sampling sites, these were not retrievable the following sampling season. Individuals were acclimated to laboratory conditions in flow-through channels (168 h; 8, 9 or 11°C for springs S1, S2 and S3, respectively, based on the temperatures measured at each spring during sampling). Thirty-one individuals per population were then randomly divided in four temperature treatment groups (for 168 h; 8, 11, 14 or 17°C). After this period, individuals were placed in a custom-built oxygenated water bath equipped with a plastic plate containing 2 ml glass vials, with one animal per glass vial. Temperature and dissolved oxygen (always >90%) were monitored throughout each experiment (MultiLine Multi 3650 IDS). The water bath was started at 8°C and heated up continuously to 34°C (0.25°C min^−1^) using a flow-through water heater (Hydor T08100) controlled by a proportional integral derivative controller (SYL-2352P) via a solid state relay and a K-type thermocouple. We recorded the CT_max_ value for each individual at the point at which it first curled up and arched its body according to Claussen and Walters (1982).

### Proteomics experiment

In a separate experiment, we sampled individuals of similar size (*n*=100) from the alpine population (August 2020; spring S3 in Fig. S1) and acclimated them for 168 h at 8°C to laboratory conditions. After 168 h, individuals were randomly placed in 8, 11, 14 and 17°C (±0.25°C s.d.) flow-through channels for an additional 168 h. We chose these temperatures as they reflect predicted temperature increases in the Swiss Alps, with 8°C being the control temperature that was measured at the time of sampling, 11°C the IPCC high emission scenario RCP8.5 until mid-21st century, 14°C the RCP8.5 scenario for the end of the 21st century in Switzerland and 17°C an outlier temperature thought to elicit a clearly detectable signal at the proteome level, to better characterize *C. alpina*'s proteome responses to warming. For each treatment temperature, 5 biological replicates (3 animals of similar size pooled per replicate) were kept in separate oxygenated boxes. Temperature, pH, conductivity and oxygen were monitored throughout the experiments and animals were reared on a light (L):dark (D) cycle of L10 h:D14 h. Animals were starved throughout the experiment and each box contained filtered (0.45 µm pore size) water obtained from the spring. At the end of the experiment, individuals were collected with a 2 ml pipette, blotted dry on filter paper, washed with 1× PBS, transferred to 1.5 ml protein LoBind tubes (Eppendorf), shock-frozen in liquid nitrogen and stored at −80°C until further processing.

### Proteome analysis

Samples were thawed on ice and whole organisms treated with 5% *N*-acetyl-l-cysteine (NAC; Sigma-Aldrich) in 1× PBS for 8 min on ice to remove their mucous coating (Pearson et al., 2009). Samples were vortexed and spun down at 5000 rpm for 2 min (at 4°C) and the NAC decanted. Lysates were generated from whole organisms by adding 150 µl lysis buffer [5% SDS, 10 mmol l^−1^ TCEP, 100 mmol l^−1^ ABC, supplemented with 1 mmol l^−1^ Halt^TM^ protease inhibitor cocktail (Thermo Scientific)], homogenizing samples manually with a glass pestle and subjecting them to 10 cycles of ultrasonication (30 s on, 30 s off) using a Bioruptor Pico at 4°C (Diagenode, Inc.). To denature proteins, samples were heated for 10 min at 95°C and mixed at 300 rpm in a PCR 96 thermomixer. At this point, protein concentrations were determined via a bicinchoninic acid assay (BCA; Thermo Scientific Pierce) according to the manufacturer's instructions (Table S1). After letting samples cool down to room temperature (RT), they were spun down at 5000 rpm for 10 s, 0.5 µl iodoacetamide (1 mol l^−1^) was added and samples were kept in the dark at 25°C for 30 min at 500 rpm. Further analytical steps were performed with an aliquot of no more than 50 µg protein. Following a suspension trapping (S-Trap) protocol (HaileMariam et al., 2018), 2.5 µl 12% phosphoric acid and 165 µl of S-trap buffer (90% methanol, 100 mmol l^−1^ TEAB, pH 7.1) were added to each sample. Samples were transferred to S-trap micro-columns and washed 3 times by adding 150 µl S-trap buffer followed by centrifugation at 4000 ***g*** for 1 min at each wash. Columns were then placed in fresh 2 ml tubes and 20 µl digestion buffer (50 mmol l^−1^ TEAB, pH 8), supplemented with 1:25 trypsin (Sequencing Grade Modified Trypsin, Promega), was added to each column. Matrix-bound proteins were digested at 47°C for 1 h and the resulting peptides collected by adding 40 µl of digestion buffer to columns and spinning at 4000 ***g*** for 1 min. Next, 40 µl of 0.2% formic acid was added to each column, followed by centrifugation at 4000 ***g*** for 1 min, then 35 µl of 50% acetonitrile containing 0.2% formic acid was added, and spun down at 4000 ***g*** for 1 min. Eluted peptides were concentrated to dryness by applying a vacuum for 2 h. Peptides were subsequently dissolved in 20 µl 0.1% formic acid by 10×1 s ultrasonication and shaking at 1400 rpm at 25°C for 5 min. Peptide concentrations were determined based on absorbance values using a SPECTROstar Nano Absorbance Plate Reader (BMG Labtech). Peptides were diluted to a concentration of 0.5 µg µl^−1^ in LC-buffer A. IRT peptides (Biognosys AG, Schlieren, Switzerland) were added to control for LC-MS performance, and samples were stored at −20°C prior to LC-MS/MS analysis using an Orbitrap Fusion Lumos Tribrid Mass Spectrometer fitted with an EASY-nLC 1200 (both Thermo Fisher Scientific) and a custom-made column heater set to 60°C. Peptides were resolved using an RP-HPLC column (75 µm×36 cm) packed in-house with C18 resin (ReproSil-Pur C18-AQ, 1.9 µm resin; Dr Maisch GmbH) at a flow rate of 0.2 µl min^−1^. The following gradient was used for peptide separation: from 5% B to 12% B over 10 min to 35% B over 80 min to 50% B over 30 min to 95% B over 2 min followed by 18 min at 95% B. Buffer A was 0.1% formic acid in water, and buffer B was 80% acetonitrile, 0.1% formic acid in water. The mass spectrometer was operated in data-dependent acquisition (DDA) mode with a cycle time of 3 s between master scans. Each master scan was acquired in the Orbitrap at a resolution of 120.000 full width at half maximum (at 200*m*/*z*, MS1) and a scan range from 375 to 1.600*m*/*z* followed by MS/MS (MS2) scans of the most intense precursors in the linear ion trap at ‘Rapid’ scan rate with isolation of the quadrupole set to 1.4*m*/*z*. Maximum ion injection time was set to 50 ms (MS1) and 35 ms (MS2) with an AGC target of 1.0E6 and 1.0E4, respectively. Monoisotopic precursor selection (MIPS) was set to peptide, and the intensity threshold was set to 5.0E3. Peptides were fragmented by HCD (higher-energy collisional dissociation) with collision energy set to 35%, and one microscan was acquired for each spectrum. The dynamic exclusion duration was set to 30 s.

### Protein identification and data analysis

Raw MS/MS spectra were converted to mzML format using MSConvert (Adusumilli and Mallick, 2017) and subsequently submitted to a closed search with default parameters in the MSFragger (Kong et al., 2017) v.3.1.1 pipeline as implemented in FragPipe v.14.0 (https://fragpipe.nesvilab.org). Spectra were matched against a deduced *C. alpina* transcriptome with the addition of common laboratory contaminants. The transcriptome *de novo* assembly pipeline was identical to the one used for other planarian transcriptomes available from the PlanMine database (Rozanski et al., 2019). From the *de novo* assembled transcriptome, we extracted long open reading frames (ORFs) using the TransDecoder v.5.3.0 pipeline TransDecoder.LongOrfs followed by TransDecoder.Predict. We specifically identified ORFs with homology to known proteins in the Pfam database (El-Gebali et al., 2019) with HMMER (Durbin et al., 1998) v.3.2.1 and subsequently used TransDecoder.Predict with the --retain_pfam_hits option specified. Redundancy of sequences in the deduced proteome was reduced using CD-hit (Li et al., 2001) v.4.8.1 with an identity cut-off of 0.9 and a minimum word size of 5. In MSFragger, enzyme specificity was set as fully tryptic, with a maximum of two missed cleavages. The peptide spectrum-match false discovery rate (FDR) and the protein FDR were both set to 0.01 (based on the target-decoy approach) using the Philosopher toolkit v.3.3.12 (da Veiga Leprevost et al., 2020). Precursor mass tolerance was set to 50 ppm and fragment mass tolerance was set to 20 ppm, with mass calibration and parameter optimization enabled. Label-free quantification (LFQ) was performed using IonQuant (Yu et al., 2021) and the match between runs option (MaxLFQ algorithm; minimum ions: 2). Oxidation of methionine (M) and acetylation (Protein N-term) were specified as variable and carbamidomethylation of cysteines (C) as fixed modifications. Minimum peptide length was set to 7 amino acids with two allowed missed tryptic cleavages. From the MSFragger protein-level output ‘reprint.int.’, the razor intensity columns (‘unique+razor’) were used for downstream analyses in R v.4.0.2 (http://www.R-project.org/). LFQ intensities had ∼19.95% missing values which were imputed using DreamAI, an ensemble machine learning algorithm specifically designed for LFQ data (Ma et al., 2020 preprint). Imputed relative abundances were then subjected to variance stabilization normalization (VSN) using the function normalizeVSN in the Linear Models for Microarray Data (LIMMA) package v.3.46.0 (Smyth, 2005). This method has been shown to outperform other normalization strategies in label-free proteomics data (Välikangas et al., 2018). Unsupervised non-metric multidimensional scaling (nMDS) was performed to visualize proteome differences following acclimation. For this, a Bray–Curtis distance matrix was computed based on relative abundance for each protein and used as input to the metaMDS function in vegan v.2.5.7 (https://CRAN.R-project.org/package=vegan). To identify differentially abundant proteins (DAPs), we computed pairwise differences using the LIMMA package. A linear model was fitted for each protein via function lmFit and contrasts from the model fit and summary statistics were computed via the functions contrasts.fit and eBayes. A list of significant proteins was generated using the function topTable, and *P*-values were adjusted for multiple testing using the Benjamini–Hochberg (BH) procedure. After testing for normality and homogeneity of variance, one-way analyses of variance (ANOVA) followed by *post hoc* Tukey Tests were performed on selected proteins. Suites of co-abundant proteins were identified using two methods. (1) For a soft clustering approach, we averaged protein LFQs over the 5 biological replicates per treatment, standardized the data (mean=0, s.d.=1) and subjected them to the fuzzy c-means algorithm as implemented in the Mfuzz (Kumar and Futschik, 2007) package v.2.50.0 with a fuzzification parameter of 2.54 and 8 centres (Fig. S2B,H). (2) Weighted Correlation Network Analysis (WGCNA) v.1.69 was used to identify networks of functionally co-regulated protein groups (Langfelder and Horvath, 2008). First, a sample network was constructed to identify outlying samples with a standardized connectivity score of less than −2.5 (Horvath, 2011). A signed protein co-abundance network (minModuleSize=30) was constructed with a soft threshold power (β) of 12 as found appropriate by the function pickSoftThreshold to reach a scale-free topology index of at least 0.90. We used the Dynamic Tree Cut approach to merge highly correlated modules using a height-cut of 0.25 (Zhang and Horvath, 2005). Module eigengenes (the first principal component of the abundances of all proteins in a module across replicates) were correlated with acclimation temperature as a continuous variable (Fig. S2C–G).

### Proteome annotation

All MS/MS-identified (*n*=3399) protein sequences were queried against the UniProtKB/SwissProt database using stand-alone blastp v.2.11.0 (max_target_seqs=10, minimum e-value=0.000001). Matches with the lowest e-value were extracted from the results and sequences with no hits to the SwissProt database (*n*=282) were queried against the TrEMBL database with parameters as above. Remaining, unmatched sequences (*n*=122) were queried against the NCBI non-redundant database. When a sequence had no hits to any of the three databases (*n*=86), we performed structure homology modelling using SWISS-MODEL (Waterhouse et al., 2018). Protein domains were determined for all sequences using stand-alone InterProScan v.5.39.77.0 with default parameters (Jones et al., 2014). All sequences were assigned to Clusters of Orthologous Groups (COGs) (Tatusov et al., 2000), Kyoto Encyclopedia of Genes and Genomes orthology terms (KO) (Kanehisa and Goto, 2000) and Gene Ontology (GO) terms in eggNOG (Huerta-Cepas et al., 2019) v.5.0 via eggNOG-Mapper v.2 (Taxonomic scope: automatic, Orthologs: all orthologs, GO evidence: non-electronic terms, E-value: 0.001, minimum hit bit-score: 60). We used SignalP (Almagro Armenteros et al., 2019) v.5.0 and TMHMM (Krogh et al., 2001) v.2.0 to identify excreted proteins and transmembrane proteins. Finally, all annotation results were merged into a consensus annotated proteome (available as SI2 in Dryad: doi:10.5061/dryad.dfn2z3541). To test whether any GO terms were over-represented in mFuzz clusters and WGCNA modules, we sorted proteins by their membership and gene significance values, and performed ranked-based tests for each assigned GO term by applying Kolmogorov–Smirnov tests via package topGO (https://bioconductor.org/packages/topGO/) v.2.42.0, with the *C. alpina* proteome as the GO background universe (method=‘weight01’). We then used the clusterProfiler (Yu et al., 2012) v.3.12 to perform KO enrichment analyses (proteins with membership value >0.5; Benjamini–Hochberg adjusted *P*<0.05) and visualized results using package enrichplot (Yu, 2020) v.11.2, again using the whole *C. alpina* proteome as the KO background universe. To corroborate and expand upon GO enrichment results, we performed domain-centric GO enrichment of clusters and modules using the dcGO (Fang and Gough, 2013) suite with a stringent FDR of <0.005 and the IPS-predicted Pfam terms as input. For semantic summarization, we visualized dcGO enrichment results of clusters and modules through treemaps using REVIGO (Supek et al., 2011) with the SimRel semantic similarity measure and a medium allowed similarity of 0.7. As functional enrichment analyses [i.e. (dc)GO and KO enrichment] are inherently biased (Timmons et al., 2015; Tomczak et al., 2018), we additionally scanned the literature individually for each DAP to deduce their functional roles.

## RESULTS

**Fig. 1.**
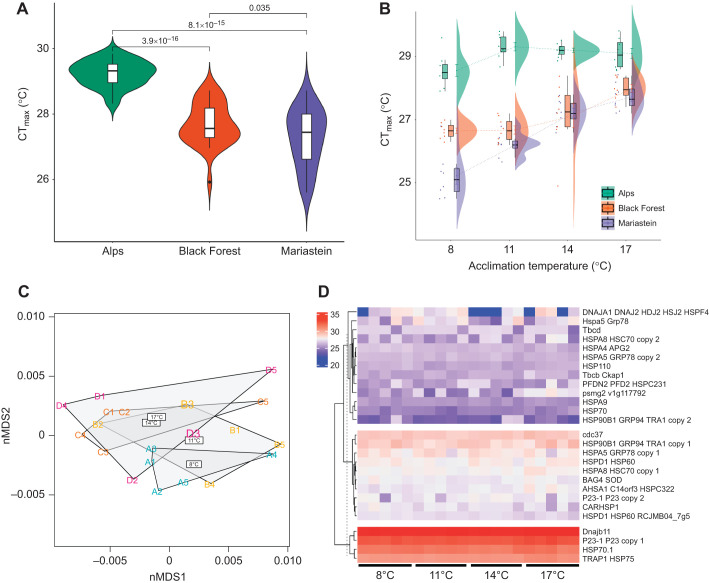
**Critical thermal maximum (CT_max_) and proteomics results.** (A) Violin plots of CT_max_ values of three *Crenobia alpina* populations (*n*=31 per population; see Fig. S1 for information on sites) independent of acclimation temperature. *P*­-values are shown above the brackets (ANOVA). (B) Raincloud plot of CT_max_ values following thermal acclimation of the three *C. alpina* populations shown in A. (C) Non-metric multidimensional scaling (nMDS) ordination based on the relative abundance of 3399 proteins commonly identified across *n*=5 biological replicates per acclimation temperature in the alpine population (Alps) following 168 h acclimation to 8, 11, 14 or 17°C. (D) Heatmap of all heat shock proteins (HSPs) quantified in the alpine population's proteome following 168 h thermal acclimation to 8, 11, 14 or 17°C. Colours represent the log_2_ label-free quantification (LFQ) values for each HSP measured in the 20 biological replicates of the alpine *C. alpina* population (columns; *n*=5 replicates per acclimation temperature).

**Fig. 2.**
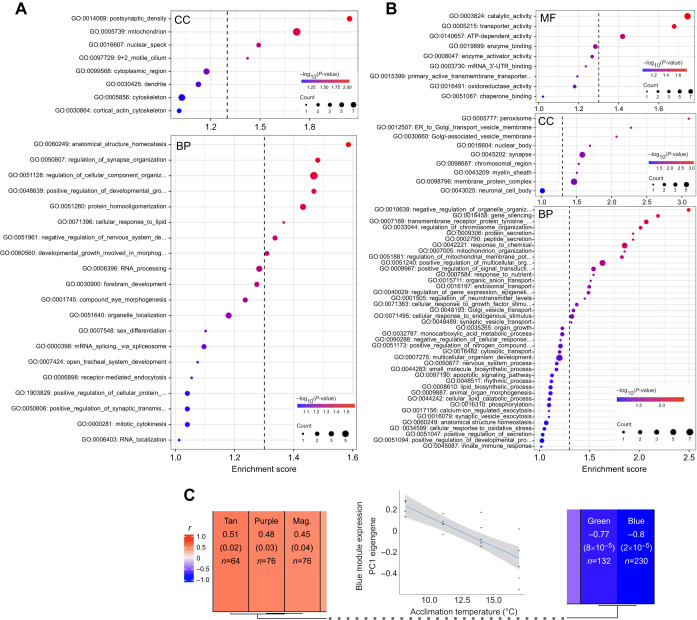
**Proteome changes of *C. alpina* following 168 h acclimation to sublethal temperatures.** (A) GO enrichment results of proteins of higher abundance following acclimation (WGCNA modules: Tan, Purple and Magenta) for the Cellular Compartment (CC; top) and Biological Process (BP; bottom) GO category. (B) GO enrichment results of proteins of lower abundance following acclimation (WGCNA modules: Green and Blue) for the Molecular Function (MF; top), Cellular Compartment (CC, middle) and Biological Process (BP, bottom) GO category. Dashed lines indicate an adjusted *P*-value cut-off of 0.05. Colours represent the adjusted *P*-value and dot size indicates the number of significant proteins associated with the respective GO term. (C) Names (top) and statistics of WGCNA modules and their association with acclimation temperature, represented by Pearson's correlation coefficients (*r*) between module eigengene (ME) and acclimation temperature, the *P*-value of the correlation in parentheses and the number of proteins assigned to each module (*n*; with membership value >0.5). The plot in the middle shows the correlation between the ME expression of the Blue module and acclimation temperature.

CT_max_ was 1.46°C higher in the alpine *C. alpina* population (Fig. 1A), which showed reduced CT_max_ plasticity following thermal acclimation (Fig. 1B). Differences in organism size may have had an influence (Dallas and Rivers-Moore, 2012), but no significant effect was found (Table S2). The mean (±s.d.) CT_max_ of all individuals, independent of source population, was 27.58±1.34°C (*n*=96), which is within the range of other planarian species and outweighs the species' theorized thermal niche maximum of 15°C (Claussen and Walters, 1982; Everatt et al., 2014). LC-MS/MS of the alpine population following acclimation resulted in an average of 120.507±3264 acquired spectra per sample, mapping to 12.677±1063 *in silico* digested peptides. All samples collectively covered 10.74% (3399 out of 31,653 predicted ORFs) of the transcriptome *de novo* assembly. Protein abundance profiles separated between acclimation groups in the nMDS space, whereby the 8°C centroid was furthest from that for 17°C, followed by 14°C and 11°C, indicating unique proteome states following acclimation (Fig. 1C). However, the separation between treatments was marginal, indicating overall proteome stability within the studied temperature range. C-means fuzzy clustering identified eight unique clusters with varying abundance patterns, ranging from linearly decreasing to stepwise increasing (Fig. S2H). WGCNA assigned all proteins to 15 co-abundance modules and six module eigengenes significantly (*P*<0.05) correlated with acclimation temperature. Both the ‘green’ (*r*=0.77) and ‘blue’ (*r*=0.8) modules correlated negatively, and modules ‘tan’ (*r*=0.51), ‘purple’ (*r*=0.48) and ‘magenta’ (*r*=0.45) correlated positively with acclimation temperature (Fig. 2B,C). These protein sets were enriched for multiple GO and KO terms (Fig. 2) and contained numerous proteins higher and lower in abundance mediating various cellular and physiological processes that changed in response to prolonged thermal acclimation and which are described in detail below.

### Decreased protein abundance

#### Oxidative stress

Acclimation to higher temperatures resulted in a decreased abundance of proteins regulating cellular responses to oxidative stress. The blue and green WGCNA modules were enriched for GO terms ‘cellular response to oxidative stress’ (GO:0034599), ‘oxidation-reduction process’ (GO:0055114), ‘regulation of reactive oxygen species metabolic process’ (GO:2000377) and ‘peroxisome’ (GO:0005777). In mFuzz cluster 3, ‘aldehyde dehydrogenase NAD+’ (K00128) was an enriched KO term, and ‘reactive oxygen species biosynthetic process’ (GO:1903409) and ‘superoxide metabolic process’ (GO:0006801) were enriched GO terms. Proteins associated with these functions were cat, prdx6, bco2, sod1, sod2, aldh1b1 and aldh3a2.

#### Protein synthesis

Proteins regulating translational control and ribosome biogenesis showed a lower abundance following warm acclimation. Enriched KO terms were ‘large subunit ribosomal protein L6e’ (K02934) and ‘endoplasmic reticulum chaperone BiP’ (K09490). Enriched GO terms included ‘translation’ (GO:0006412), ‘translational initiation’ (GO:0006413), ‘translational elongation’ (GO:0006414), ‘ribosome assembly’ (GO:0042255), ‘ribosomal small subunit biogenesis’ (GO:0042274), ‘endoplasmic reticulum (ER) stress-induced pre-emptive quality control’ (GO:0061857) and ‘protein localization to ER’ (GO:0070972). Proteins related to these terms were multiple 40S and 60S ribosomal subunits (rps3-a, rps8, rps13, rps14, rps23, rpl3, fpl4, rpl6, rpl21, rpl14, rpl18), 60S acidic ribosomal proteins (fplp0, rpp1b, rpp-2), the ubiquitin-60S ribosomal protein L40 (rpl40) and multiple eukaryotic translation initiation factors (eif3d, eif3m, eif3i, infB, eif1ax, eif-5A, eif4h, elf-4g).

#### Cellular transport

Proteins related to cellular transport were also over-represented in those with decreased abundance following warm acclimation. ‘Transporter activity’ (GO:0005215), ‘endosomal transport’ (GO:0016197), ‘ER to golgi transport vesicle membrane’ (GO:0012507) and ‘golgi-associated vesicle membrane’ (GO:0030660) were enriched terms in the blue and green WGCNA modules (Fig. 2B). Additionally, ‘small guanosine triphosphatase’ (GTPase) was the most frequent Pfam term in mFuzz cluster 3 and both the blue and green modules with associated enriched KO terms ‘RHOC’ (K07857) and ‘RHOA’ (K04513). These transport proteins facilitate endosome to Golgi transport (snx2, snx12), protein transport into the nucleus (ntf2), transport across the ER (sec24b, sec61b, tram1l1) and the retention of ER resident proteins (ssr1, ssr2).

#### Muscle contraction

Proteins regulating muscle contraction and constitution had a lower abundance following warm acclimation. GO terms ‘muscle system process’ (GO:0003012), ‘muscle attachment’ (GO:0016203), ‘muscle cell cellular homeostasis’ (GO:0046716), ‘striated muscle tissue development’ (GO:0014706) and ‘regulation of muscle hypertrophy’ (GO:0014743) were enriched in warm-responsive clusters and modules. Additionally, transgelin (K20526) was an enriched KO term in cluster 3, represented by cnn1, myophilin (N/A) and tagln3. Lower abundance proteins regulating muscle composition and contraction were unc-89, tni-4, tnt, tropomyosin A, two copies of d-titin (sls), ankyrins (ank2, ank3) and multiple collagens (col4a1, col27a1b, col1a2).

#### Regeneration

Two proteins with high sequence similarity to *Schmidtea mediterranea* smedwi-1 and smedwi-2 (72.3% and 75.7% respectively) were of lower abundance following warm acclimation. These proteins belong to the PIWI/Argonaute family and participate in the synthesis and function of small RNAs called PIWI-associated RNAs (piRNAs) which regulate neoblast function and, consequently, regeneration (Reddien et al., 2005a,b; Palakodeti et al., 2008). In addition, the heat shock protein (HSP) 40 family member dnaja1 was also of lower abundance, a chaperone required for the stability of smedwi-1 and smedwi-2 (Wang et al., 2019).

#### Mitochondrial dynamics

The GO terms ‘ATP-dependent activity’ (GO:0140657), ‘mitochondrion organization’ (GO:0007005) and ‘regulation of mitochondrial membrane potential’ (GO:0051881) were enriched in the green and blue modules. Proteins associated with these GO terms were components of vacuolar ATPase (vha26, vha14, vha68-2, atp6v1c1a, vhaac39-1, vha55, nkb-3), ATP synthase (atpsyncf6, atp5 mg, atpsynd) and ATPase (serca, nkb-1, nkb-3). Additionally, a mitochondrial 10 kDa HSP (cisd3) and a MICOS complex subunit (hnaj_locus369) were lower in abundance following warm acclimation.

### Increased protein abundance

#### Wound healing

Proteins involved in haemostasis, the first stage of wound healing, were of higher abundance following warm acclimation as indicated by the enriched GO terms ‘platelet activation’ (GO:0030168), ‘regulation of response to wounding’ (GO:1903034) and ‘positive regulation of wound healing’ (GO:0090303). Proteins mediating these processes were pdcd10, septin5, vwf, hspg2 and gp5.

#### Cilia-related proteins

Cilia-related proteins had higher abundance following warm acclimation as indicated by enriched of the GO term ‘9+2 motile cilium’ (GO:0097729) in the CC category of warm-responsive WGCNA modules (Fig. 2A). The proteins dctn1, pdcl, ttc26, ift80, mks1, cfap70 and ttc21b were of higher abundance following warm acclimation, and so were multiple dynein proteins such as a dynein beta chain (ciliary N/A), dynlrb2, dnah2 and two copies of dnah5.

#### Developmental growth

The GO terms ‘anatomical structure homeostasis’ (GO:0060249), ‘positive regulation of developmental growth’ (GO:0048639) and ‘developmental growth involved in morphogenesis’ (GO:0060560) were enriched in the warm-responsive WGCNA modules (Fig. 2A). Higher-abundance proteins were involved in three associated processes: nervous system development (otk, alpha-Spec, manf, emb-9, prss12, futsch, hil), cell division and fate determination (myh9, cdtn1, ist1, snx33, par-5, flil, cdc73, def6), and photoreceptor function/eye development (rab18, neur, slc2a1).

#### Immune system

Immune system processes were over-represented in warm-responsive clusters and modules; for example, GO terms ‘immune system process’ (GO:0002376), ‘immune response’ (GO:0006955) and ‘myeloid leukocyte mediated immunity’ (GO:0002444) in the magenta module. Associated proteins mx2, dock2, mrc1, sftpd, ifih1, ikbke, atg101, dpp9, rbck1 and mpeg1 showed increased abundance compared with the control temperature proteome state.

#### Muscle contraction

In contrast to proteins that positively regulate muscle contraction, proteins that negatively regulate contraction and differentiation such as unc-22, unc-89, crvp, csrp2 and a ryanodine receptor (ryr) exhibited increased abundance following warm acclimation. Additionally, myoglobin was of higher abundance, a protein that facilitates the movement of oxygen within muscles (Wittenberg, 1970).

#### Mitochondrial dynamics

While proteins associated with ATPases and ATP synthases were lower in abundance following warm acclimation, higher-abundance proteins were enriched for mitochondria (GO:0005739; Fig. 2A). These include proteins forming complexes of the electron transport chain: subunits of NADH dehydrogenase [dea37_0010400 (beta subcomplex subunit 6), d2030.4 (beta subcomplex subunit 7), NDUFA13 (alpha subcomplex subunit 13), ND-42 (alpha subcomplex subunit 10)], subunits of succinate dehydrogenase [sdhb (iron-sulfur subunit), sdhc (b560 subunit)], and a subunit of cytochrome bc1 (cyc1). Additionally, two voltage-dependent anion-selective channel 2 proteins (cdac2), and mitochondrial transmembrane transporter proteins (2× sh3glb1, tim14) were of higher abundance following warm acclimation.

## DISCUSSION

### CT_max_ and HSPs

The ∼28°C CT_max_ of *C. alpina* indicates short-term tolerance to warming temperatures and the three populations exhibited both CT_max_ and CT_max_ plasticity differences, whereby the alpine population exhibited the highest and least plastic CT_max_. While these results suggest acute higher thermal tolerance for this species, CT_max_ measurements are not necessarily good predictors of long-term acclimation capacity, and depend on both the ramping rate and the starting temperature of the assay (Terblanche et al., 2007; 2011). Following long-term acclimation, individuals of the alpine *C. alpina* initiated an unfolded protein response (UPR), characterized by a higher abundance of HSP90 co-chaperones cdc37 and unc45a, ube2j1, nap1l4, dnaj homolog dnajc3, three disulfide-isomerases (2×pdi-2, p4hb), sdf2, ppib and tmbim6. Interestingly, all identified HSPs were of comparable abundance following acclimation (Fig. 1D), a pattern that may be due to increasing levels of cold denaturation through the hydrophobic effect (Tsai et al., 2002). Alternatively, while HSPs are initially induced upon a temperature increase, their expression reduces if high temperatures persist, partly because chronic induction can lead to detrimental effects in cells (Gasch et al., 2000; Wang et al., 2006; Roth et al., 2014). The initiation of an UPR and a high number of identified HSPs suggests that *C. alpina* did not lose mechanisms to assist refolding or removing damaged proteins should temperatures increase, separating it from certain cold-adapted species from the frigid (e.g. Hofmann et al., 2000; Clark et al., 2008) or temperate zones (e.g. Bosch et al., 1988).

### Mitochondrial respiration and oxidative stress

Adjustments in mitochondrial properties and capacity are essential features of temperature acclimation in a variety of species. In *C. alpina*, warm acclimation resulted in increased abundance of electron transport chain (ETC) complex subunits, but in lowered abundance of ATP synthase and ATPase subunits. These results suggest an increase in mitochondrial respiration at warmer temperatures, while simultaneously indicating decreased levels of ADP phosphorylation and ATP dephosphorylation. Alternatively, lowered amounts of ATP synthases and ATPases may reflect an adaptive mechanism that limits ATP use for non-essential pathways, or indicate that the activity of these proteins is increased at higher temperatures such that lower numbers are needed to compensate for reduced enzyme activity at lower temperatures. Additionally, elevated mitochondrial respiration commonly leads to an increased production of reactive oxygen species (ROS; Abele et al., 2002). However, for *C. alpina*, we observed a lowered abundance of proteins mitigating oxidative stress following warm acclimation. Previous studies identified an increase in oxidative stress proteins during cold acclimation (Mádi et al., 2003; Ibarz et al., 2010), in particular of superoxide dismutases sod1 and sod2 (Malek et al., 2004). Taken together, these findings suggest complex responses at the proteome level regarding mitochondrial responses and that oxidative stress for *C. alpina* may be higher at low compared with high temperatures.

### Cellular transport

Exposure to cold leads to a depression in the rate of virtually all biological processes, including transport mechanisms which regulate cellular functioning (Kuismanen and Saraste, 1989; Malan and Canguilhem, 1989). Multiple Rab homologues, sorting nexins and nuclear transport factors were of lower abundance following thermal acclimation, proteins which function as regulators of transportation networks (Novick and Zerial, 1997; Cullen, 2008). Proteins from the Rab family, for example, regulate intracellular vesicular transport and trafficking of proteins between organelles (Zerial and McBride, 2001; Etienne-Manneville and Hall, 2002). An extensive study on the effects of prolonged thermal acclimation on the proteome of *Saccharomyces cerevisiae* showed that protein re-localization is a core cellular response (Domnauer et al., 2021) and transport proteins have commonly been found to be up-regulated following warm acclimation (Palumbi et al., 2014; Veilleux et al., 2015). As in the case of a lower abundance of proteins functioning in protein synthesis (see below), it is difficult to discern whether this response constitutes a disruption of cellular functioning or whether lesser amounts of these proteins are needed as a result of the absence of cold-induced repression of transportation. The latter scenario leads us to hypothesize that higher temperatures may reduce the need to compensate cellular transport functioning in *C. alpina* at cold temperatures, in turn leading to reduced demands on this core cellular process.

### Protein synthesis

In *C. alpina*, a lower abundance of 15 identified ribosomal subunits and 8 identified translation initiation factors following warm acclimation suggests reduced protein synthesis at warmer temperatures. As repression of protein synthesis is a feature of cells exposed to elevated temperatures (Duncan and Hershey, 1989), attributed to inhibition of translation initiation (Shalgi et al., 2013), this down-regulation may ensure the conservation of resources that are needed to survive adverse conditions (Sheikh and Fornace, 1999; Larade and Storey, 2007). However, lower temperatures slow down RNA translational efficiency, and reduce *in vivo* protein synthesis (Friedman et al., 1971; Broeze et al., 1978; Fraser et al., 2002b). A lowered abundance of these proteins at higher temperatures may therefore reflect a reduced need to compensate for impaired protein synthesis at colder temperatures. These findings lead to an interesting question: in species from cold-stable environments, does a lowered abundance of proteins functioning in the protein synthesis machinery represent a ‘positive’ (i.e. reduced demand at higher temperatures) or ‘negative’ (i.e. impaired protein synthesis) response to elevated temperatures?

### Reduced DNA repair and demands on molecule structural flexibility

Mechanisms such as alternative splicing and control of nucleic acid topology allow organisms to alter molecule thermostability (López-García and Forterre, 2000; D'Amico et al., 2002). At colder temperatures, RNAs fold into thermodynamically stable secondary structures which impede transcription and translation. In *C. alpina*, three Y-box homolog proteins containing a cold-shock domain (CSD) were of lower abundance following warm acclimation (Fig. S3). The accuracy and regulation of alternative splicing events is also temperature dependent (Hopkins et al., 2018; Healy and Schulte, 2019), and multiple splicing-related proteins were of lower abundance at higher acclimation temperatures. These include srsf1, a protein that prevents exon skipping (Kohtz et al., 1994), nova1, rbm22, khsrp, dhx8, two copies of khdrbs2, ddx21 and rbm22. Additionally, a UV excision repair protein (rad23b), cbx3, an endonuclease mediating miRNA decay (snd1), serpinb10 and two 5'–3′ exonucleases (pld3, xrn1) were lower in abundance following warm acclimation, indicating a reduced need to mitigate cold-induced DNA damage and topology restrictions at higher temperatures. Based on these findings, we hypothesize that *C. alpina* maintains cold-inducible mechanisms to relax DNA topology and to ensure accurate transcript splicing, and that these processes are down-regulated at elevated temperatures.

### Proteome homeostasis

If temperature-induced damaged or misfolded proteins accumulate in the cell, they may become cytotoxic and interfere with cellular function (Sherman and Goldberg, 2001). If proteins are beyond repair, they are degraded by proteases, primarily by the ubiquitin–proteasome pathway (Wickner et al., 1999). In *C. alpina*, ubiquitin-related proteins (ubq11, rpL40, ubcD1, ube2l3, uba1 and ube2z) and 26S proteasome-related proteins (psmd1, psmd2, psmc2, psmb2, psmb6, psmb7, psmf1, rpt6 and rpn11) were of lower abundance following warm acclimation. These findings indicate that changes in the ubiquitin–proteasome pathway and the degradation of ubiquitinated proteins play key roles in the maintenance of protein homeostasis at colder temperatures, but that, at higher temperatures, these processes are down-regulated in *C. alpina*. Interestingly, compared with cold-adapted *Mytilus galloprovincialis*, in warm-adapted *M. galloprovincialis*, proteasome isoforms were also shown to be down-regulated (Tomanek, 2012), potentially prolonging the lifespan of proteins implicated in coping with elevated temperatures, as has been shown for oxidative stress proteins (Bieler et al., 2009).

### Muscle contraction

The maintenance of mechanical muscle performance at low temperatures can be found in a variety of species (Bennett, 1985; Goldspink, 1995; Gillis and Tibbits, 2002; Tiitu and Vornanen, 2002; Coughlin et al., 2016) and locomotion speed commonly changes in an acclimation temperature-dependent manner (Watabe, 2002). A variety of muscle-related proteins were of lower abundance following warm acclimation of *C. alpina*. These include troponins (tnt, tni-4), which confer calcium sensitivity to striated muscle (Gomes et al., 2002), tropomyosins (tm1, smtmi and egtrpa), which additionally regulate calcium-dependent muscle contraction (Galińska-Rakoczy et al., 2008), calponins (box15_mlig001580g2, and cnn1), which modulate muscle contraction (Winder et al., 1998), and ankyrins (ank2 and ank3), which regulate muscle gene expression (Kojic et al., 2010; Laure et al., 2010). Conversely, muscle-related proteins such as unc-22, unc-45a, unc-89 and a cysteine- and glycine-rich protein 2 (csrp2) were of higher abundance following warm acclimation. These proteins facilitate sarcomere organization and extensibility, and thereby regulation of muscle composition, contraction and relaxation (Tskhovrebova and Trinick, 2003; Nyegaard et al., 2012; Koh et al., 2013). Locomotion speed of planarians is reduced at lower temperatures, and their muscle-powered movements are susceptible to temperature changes (Inoue et al., 2014). Our findings pinpoint to the proteins that are probably regulating muscle contraction and sarcomere organization during muscle adaptation to higher and lower temperatures in planarians.

### Neurotransmitter regulation and wound healing

Both dopamine and γ-aminobutyric acid (GABA) modulate temperature sensitivity (Fraser et al., 2002a; Ueno et al., 2012; Jakšić et al., 2020), and the higher abundance of proteins gch1 (Tegeder et al., 2006), manf (Palgi et al., 2009), aldh5a1 (Kim et al., 2009) and ddc (Rorsman et al., 1995) suggests regulation of the levels of these neurotransmitters following warm acclimation. Interestingly, planarian locomotion such as phototaxis is coordinated by dopaminergic and GABAergic neurons (Nishimura et al., 2007; 2008), suggesting a link between the abundance of the above-mentioned proteins and a response to warm acclimation. A higher abundance of vwf, which promotes the adhesion of platelets (Kroll et al., 1991), and gp5, which mediates vwf-dependent platelet adhesion to blood vessels (Hickey et al., 1993), suggests a possible regulation of haemostasis following warm acclimation.

### Maintenance of ciliated structures

The ventral epidermis of planarians is composed of multiciliated cells (MCCs), allowing them to glide along substrates. Specifically, cilia that populate MCCs are highly conserved, with a 9+2 axoneme and a full complement of inner and outer arm dynein motors (Rompolas et al., 2013; Azimzadeh and Basquin, 2016). Here, we found that a variety of proteins that maintain ciliated structures increased in abundance following warm acclimation. After exposure to elevated temperatures, ciliated structures were absent from the dorsal epidermis of the planarian *Girardia tigrina* (De Oliveira et al., 2018), and individuals of the planarian *Dugesia dorotocephala* exhibit reduced movement speed at higher temperatures (Claussen et al., 2003). Accordingly, we hypothesize that cilia-related proteins may increase in *C. alpina* following warm acclimation either to compensate for temperature-induced damage to MCCs or to facilitate increased movement speed at higher temperatures.

### Immune system processes

Innate immune system-related proteins showed both increased and decreased abundance following warm acclimation. Two general trends could be discerned: proteins functioning in leukotriene biosynthesis (two copies of cotl1, and lta4 h) showed reduced abundance, and proteins related to autophagy, viral infection and macrophage-like cell activity such as atg101 (Mercer et al., 2009), ikbke (Hemmi et al., 2004), ifih1 (Sarmiento et al., 2017), mx2 (Kane et al., 2013), mrc1 (Harris et al., 1992) and sftpd (Holmskov et al., 1999) showed increased abundance. Additionally, mrc1, a C-type lectin that recognizes different types of pathogens such as bacteria and fungi (Gao et al., 2017; Brown et al., 2018) was of higher abundance following warm acclimation. Together, these data indicate regulation of the innate immune system to respond to changes in the pathogenic microbial community composition following a thermal regime change. The spring water used in the acclimation experiment was filtered but microbial communities were not monitored throughout the experiment, so it cannot be ruled out that pathogens were present in higher acclimation treatments. However, temperature increase has previously been shown to increase the capacity of planarians to cope with pathogens (Hammoudi et al., 2018; Kangale et al., 2021) and cold-stable habitats such as freshwater springs harbour a unique microbial community that has only recently begun to be characterized (Savio et al., 2018; Moldovan et al., 2020). The identified changes in immune system proteins hint at unknown interactions between freshwater planarian immunity, pathogen presence and environmental temperature, providing hypotheses for future studies. For example, planarians may reduce leukotriene synthesis at warmer temperatures and increase the *de novo* synthesis of pathogen receptors and the number of macrophage-like cells. However, whether the regulation of immune system processes is related to mitigation of temperature-induced damage or is an adaptive feature of environmentally dictated pathogen resistance mechanisms cannot be gleaned from our comparative analysis alone.

### Study limitations

With regards to limitations of the proteomics approach, the *de novo* transcriptome may not contain specific transcripts that play a role in thermal acclimation as the RNA-seq data did not originate from the same individuals used for proteome analysis. Furthermore, proteins could serve purposes that have not yet been functionally annotated (e.g. moonlighting proteins), which could lead to inaccurate conclusions (Jeffery, 2014). Additionally, starvation may have had antagonistic or synergistic effects on *C. alpina*'s responses to a temperature increase compared with conditions that are not nutrient limiting. The absence of classically observed stress responses following exposure to elevated temperatures may therefore have been due to a lack of energy resulting from starvation. Future experiments should aim at comparing nutrient-limiting versus non-limiting conditions on thermal tolerance. However, the identified proteome differences between acclimation treatments should be independent of starvation-induced mechanisms as all individuals were equally starved. Finally, molecular responses to constant versus varying thermal regimes differ (Salachan and Sørensen, 2022), suggesting that molecular responses of *C. alpina* under natural conditions of warming may differ from the ones identified in this study.

### Implications for the ecology of *C. alpina*

Based on our findings, we hypothesize that *C. alpina* is able to tolerate acute and longer-term temperature increases better than previously assumed. Given that the distribution of *C. alpina* is generally restricted (Brändle et al., 2007), yet it can tolerate the typical temperatures where other planarian species occur, factors other than temperature may dictate the species' distribution (see also Vila-Farré and Rink, 2018). Interestingly, in the central Pyrenees, a single spring did not harbour more than one planarian species (Roca et al., 1992) and in Welsh streams, an exploitation competition between *C. alpina*, *Phagocata vitta* and *Polycelis felina* was observed, whereby co-existence was confined to conditions with high prey abundance (Lock and Reynoldson, 1976; Durance and Ormerod, 2010). Such competitive exclusion is frequently observed in planarians and may be the main reason for *C. alpina*'s restricted distribution. Temperature may therefore only seemingly be a limiting factor for this species, and inter-specific competition following warming of groundwater temperatures may be a stronger factor influencing future population dynamics. Finally, behavioural aspects of thermal tolerance such as phototaxis and thermotaxis, as indicated by the higher abundance of proteins related to photoreceptor development, are expected to play an important role under natural conditions (Dillon et al., 2009; Inoue et al., 2014; Paskin et al., 2014). In this context, we provide anecdotal evidence that individuals of the alpine *C. alpina* population were preferentially located on the surface of floating plants, arching their bodies towards the sun, suggesting positive phototaxis under cold-stable conditions to regulate body temperature.

### Warm tolerance of alpine aquatic species: a contentious issue?

Alpine aquatic species are considered particularly vulnerable to environmental warming because of their inability to tolerate warming temperatures (Füreder, 1999; Bernabò et al., 2011), and because of the combined effects of habitat insularity and upward distributional shifts into increasingly smaller areas of suitable habitat (Jacobsen et al., 2012; Rubidge et al., 2012; Hotaling et al., 2017). However, evidence increasingly suggests that certain high-elevation aquatic organisms may be able to tolerate warming temperatures. For example, the meltwater stonefly *Lednia tumana* and the cold-stenothermal chironomid *Pseudodiamesa branickii* exhibit high thermal tolerance, and adaptive gene expression responses to warming temperatures (e.g. expression of stable hsps and hsp70; Bernabò et al., 2011; Treanor et al., 2013; Hotaling et al., 2020), the caddisfly *Crunoecia irrorata* from cold-stable freshwater springs exhibits plastic proteome responses following thermal acclimation (Ebner et al., 2019), the cold-stenothermal chironomid *Diamesa tonsa* expresses a heat-inducible pseudo-hsp70 gene encoding a putative long non-coding RNA (lncRNA) (Bernabò et al., 2020), and, at the community level, high-elevation aquatic invertebrate communities can persist despite deglaciation (Muhlfeld et al., 2020). Despite these findings, alpine communities have also been shown to homogenize as a delayed response to increased thermal conditions (Lencioni et al., 2022). Should alpine aquatic biota indeed have a higher physiological thermal tolerance, it is reasonable to assume that factors aside from limited physiological tolerance dictate future population and community processes (see also Shah et al., 2020). While there certainly are cold-stenothermal species that are not able to tolerate warming temperatures (e.g. Schoville et al., 2015; Lencioni et al., 2022), the influence of biotic interactions at the community level such as microbial community changes (Broadbent et al., 2021), competitive exclusion (Węsławski et al., 2021), invasion potential (Holzapfel and Vinebrooke, 2005) and the reduction of available habitat (Fenoglio et al., 2010) may be dictating alpine aquatic biota persistence and restructuring under climate change scenarios.

### Conclusion

By studying how prolonged thermal acclimation affects the proteome of *C. alpina*, we have identified a variety of responses to warming temperatures in this alpine planarian. We found signs of evolved molecular cold tolerance such as CSD-containing proteins and increased abundance of proteins functioning in core cellular processes at lower temperatures (i.e. transcription, translation and cellular transport). However, an acclimation temperature-independent abundance of HSPs, an initiated UPR and a comparatively high CT_max_ lead us to suggest that *C. alpina*'s acute tolerance to warming temperatures and its acclimation capacity may historically have been underestimated. Our study indicates that this species may be a cold-adapted eurytherm rather than a cold-adapted stenotherm. CT_max_ and CT_max_ plasticity differed between populations, further suggesting that thermal history affects the species' resilience to acute temperature stress. While alpine aquatic biodiversity is adversely affected by global climate change, there is mounting evidence from molecular and community-level studies indicating that certain high-elevation, cold-stenothermal and alpine organisms may have the physiological capacity to adequately respond to warming temperatures. We suggest that other factors such as competitive exclusion may better explain their current restricted distributions. In addition, observed immune-system modulation at higher temperatures indicates largely unstudied interactions between thermal regime change, microbial composition and immune capacity of invertebrates, warranting further investigation.

## Supplementary Material

Supplementary information
