# Supplementary material for: Effects of thermal acclimation on the proteome of the planarian Crenobia alpina from an alpine freshwater spring
Source: J Exp Biol. 2022 Aug 11;225(15):jeb244218. doi: 10.1242/jeb.244218 (PMC9440759; doi:10.1242/jeb.244218)
Supplement: Supplementary information [file jexbio-225-244218-s1.pdf]

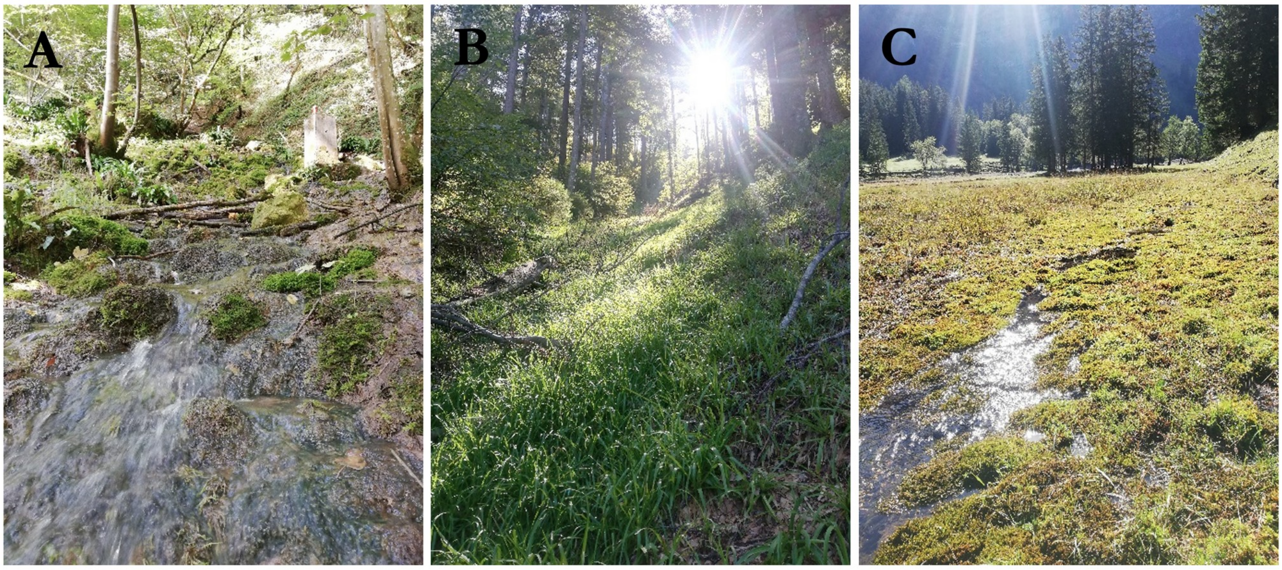

Fig. S1. Photographs of springs from which three *C. alpina* populations were taken. (**A**) Spring S1 Mariastein (Jurassic mountains; 47°28’30.8”N 7°29’34.9”E). S1 has an average temperature of 11.06 ± 0.52 °C (mean ± s.d.; averaged across three spots) with a maximum temperature of 13 °C and a minimum temperature of 9.3 °C (averaged across three spots). (**B**) Spring S2 in the Black Forest (South Germany; 47°54’27.5”N 7° 53’14.4”E). (**C**) Spring S3 in the Swiss Alps (Iffingenalp; 46°24’12.0”N 7°26’50.5”E). Spring S1 is a calcerous rheocrene spring with a mean water temperature of 11 °C and is situated 526 m.a.s.l. Spring S2 is a rheocrene spring with a mean water temperature of 9 °C situated at 1080 m.a.s.l. Spring S3 is a rheohelocrene with a mean temperature of 8 °C situated 1548 m.a.s.l.

Journal of Experimental Biology • Supplementary information

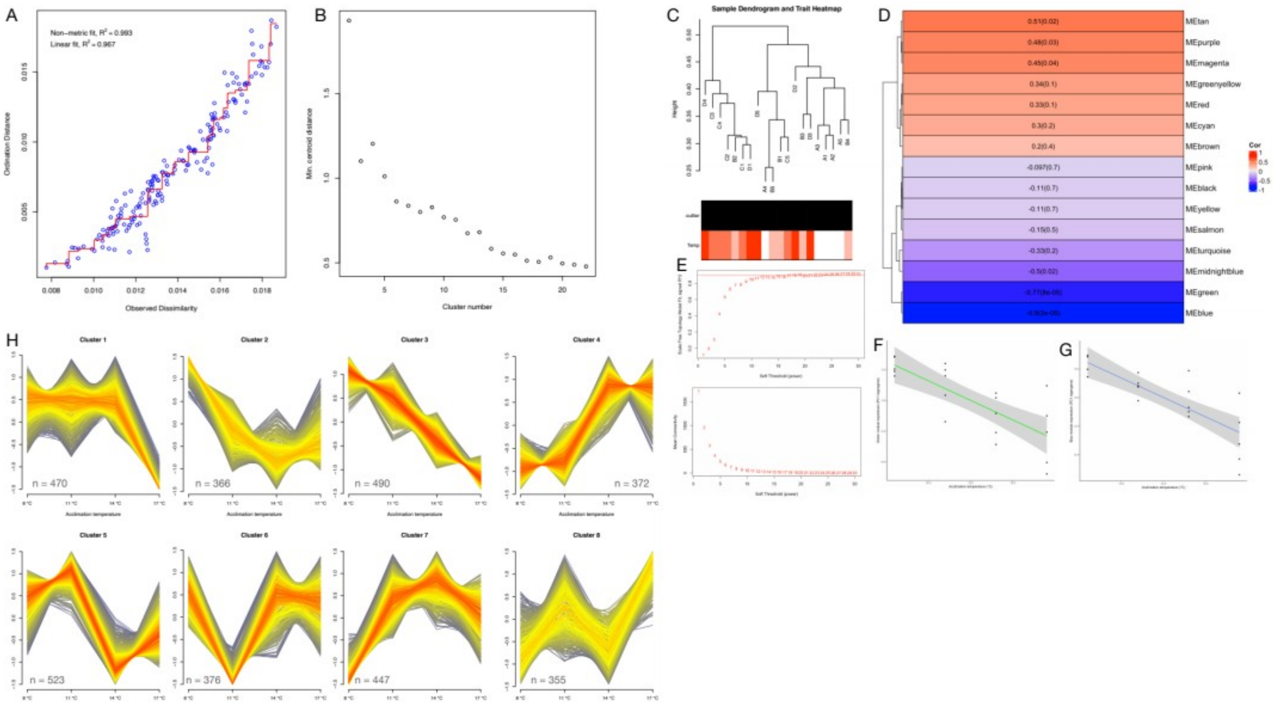

Fig. S2. Supporting statistics of (nMDS), WGCNA, and mFuzz fuzzy-set clustering. (**A**) nMDS stress-plot. (**B**) Decision plot on how many clusters (centers) to include in the mFuzz analysis (n = 8 was chosen). (**C**) Sample dendrogram showing clustering of samples based on protein abundance data, absence of outliers and sample-corresponding acclimation temperature value (white = 8, light orange = 11, orange = 15 and red = 17 °C). (**D**) Module-treatment relationships, values in cells report the correlations of the corresponding module eigengenes and acclimation temperature, with p-values printed in parentheses. The table is color coded by correlation according to the color legend. (**E**) Above plot: Analysis of global network topology for various soft-thresholding powers showing the scale-free fit index (y-axis) as a function of the soft thresholding power (x-axis). Plot below: Mean connectivity (y-axis) as a function of the soft-thresholding power (x-axis). We chose power 12 for the analysis, which is the lowest power for which the scale-free topology fit index reaches 0.90 (red line). (**F**) Linear model of acclimation temperature (°C) vs. green module eigengene expression. (**G**) Linear model of acclimation temperature (°C) vs. blue module eigengene expression. (**H**) Expression profile plots of all identified mFuzz clusters. Number of protein members per cluster are given in the lower left or right corners (no filtering applied, for enrichment analyses only proteins with a membership value > 0.5 were kept). Cluster 1 showing steady abundances until a sharp drop at 17 °C. Cluster 2 showing a U-shaped steady decrease in abundances followed by a slight rise at 17 °C. Cluster 3, showing strongly decreasing proteins over acclimation temperatures. Cluster 4 showing an increase in protein abundances. Cluster 5 showing a complex zig-zag pattern of protein abundances with a local minimum at 14 °C. Cluster 6 shows a similar abundance with a drop at 11 °C. Cluster 7 showing an increase in protein abundances followed by a stabilization and a slight decrease again at 17 °C. Cluster 8 showing unspecific membership and a complex zig-zag pattern.

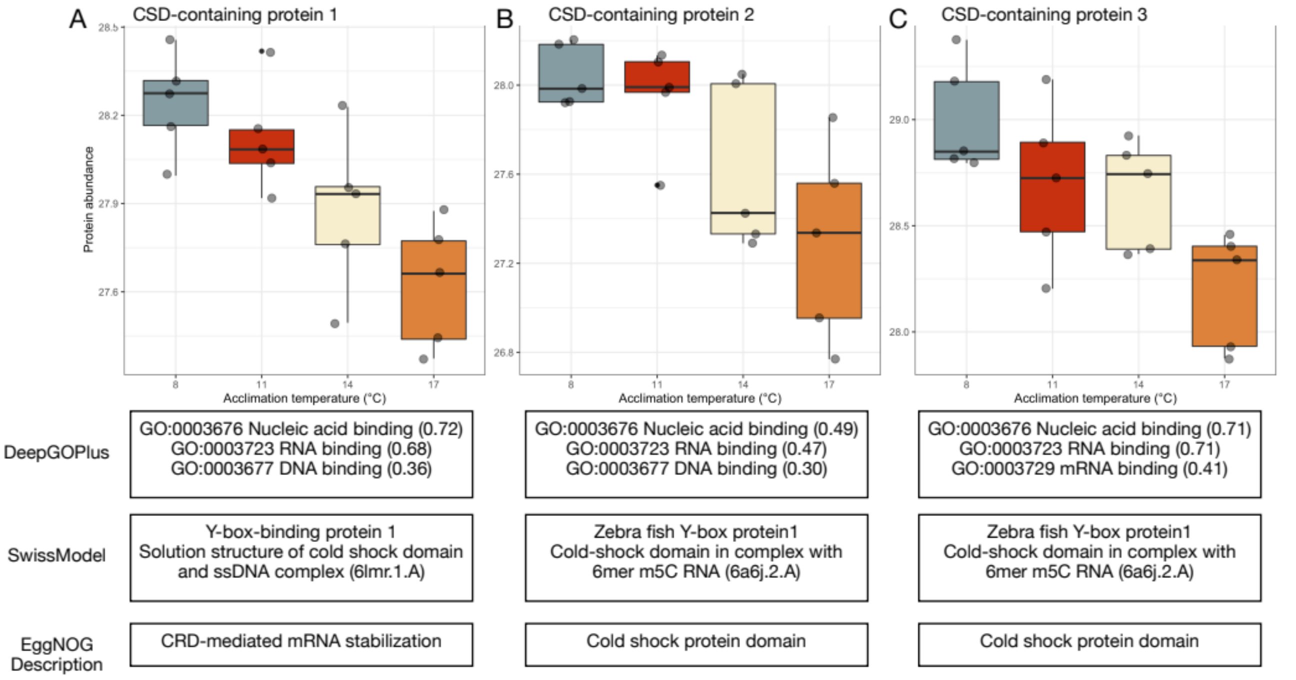

Fig. S3. Boxplots and functional annotation of three proteins containing a cold-shock domain and which were lower abundant following warm acclimation. Functional annotation is based on submitting sequences to DeepGOPlus, SwissModel and EggNOG (Waterhouse et al. 2018; Huerta-Cepas et al. 2019; Kulmanov and Hoehndorf 2020).

Table S1. Results of linear regression model with CT_{max} as response and acclimation treatment (treatment) and individual size (size; categorical) as explanatory variables (significance codes: 0 "***"; 0.001 "**"; 0.01 "*"; 0.05 "."; 0.1 "").

Term	Estimate	Standard error	T-value	P-value
(Intercept)	27.8748550	0.4925645	56.591284	<2e-16***
size	0.1878199	0.1313246	1.430196	0.1633476
treatment	0.0507826	0.0241940	2.098972	0.0446393*

References

Huerta-Cepas J, Szklarczyk D, Heller D, Hernández-Plaza A, Forslund SK, Cook H, Mende DR, Letunic I, Rattei T, Jensen LJ, et al. 2019. EggNOG 5.0: A hierarchical, functionally and phylogenetically annotated orthology resource based on 5090 organisms and 2502 viruses. Nucleic Acids Research. 47(D1):D309–D314. doi:10.1093/nar/gky1085.

Kulmanov M, Hoehndorf R. 2020. DeepGOPlus: improved protein function prediction from sequence. Bioinformatics. 36(2):422–429. doi:10.1093/bioinformatics/btz595.

Waterhouse A, Bertoni M, Bienert S, Studer G, Tauriello G, Gumienny R, Heer FT, De Beer TAP, Rempfer C, Bordoli L, et al. 2018. SWISS-MODEL: Homology modelling of protein structures and complexes. Nucleic Acids Research. 46(W1):W296–W303. doi:10.1093/nar/gky427.
